# Epigenetic Regulation of miR-212 Expression in Lung Cancer

**DOI:** 10.1371/journal.pone.0027722

**Published:** 2011-11-15

**Authors:** Mariarosaria Incoronato, Loredana Urso, Ana Portela, Mikko O. Laukkanen, Ylermi Soini, Cristina Quintavalle, Simona Keller, Manel Esteller, Gerolama Condorelli

**Affiliations:** 1 Fondazione IRCCS SDN, Naples, Italy; 2 Cancer Epigenetics and Biology Program, Bellvitge Biomedical Research Institute, 08908 L'Hospitalet, Barcelona, Catalonia, Spain; 3 Department of Pathology and Forensic Medicine, Institute of Clinical Medicine, Pathology and Forensic Medicine, School of Medicine, Cancer Center of Eastern Finland, University of Eastern Finland, Kuopio, Finland; 4 Department of Cellular and Molecular Biology and Pathology, “Federico II” University of Naples, Naples, Italy; 5 Naples Oncogenomic Center, CEINGE Biotecnologie Avanzate, Naples, Italy; 6 IEOS, CNR, Naples, Italy; 7 Facoltà di Scienze Biotecnologiche, “Federico II” University of Naples, Naples, Italy; University of Colorado Denver, United States of America

## Abstract

Many studies have shown that microRNA expression in cancer may be regulated by epigenetic events. Recently, we found that in lung cancer miR-212 was strongly down-regulated. However, mechanisms involved in the regulation of miR-212 expression are unknown. Therefore, we addressed this point by investigating the molecular mechanisms of miR-212 silencing in lung cancer. We identified histone modifications rather than DNA hypermethylation as epigenetic events that regulate miR-212 levels in NSCLC. Moreover, we found that miR-212 silencing in vivo is closely associated with the severity of the disease.

## Introduction

Worldwide, lung cancer is the most common cancer in terms of both incidence and mortality (1.35 million new cases per year and 1.18 million deaths), with the highest rates in Europe and North America. The main types of lung cancer are *small cell lung carcinoma* (SCLC) and *non-small cell lung carcinoma* (NSCLC). The non-small cell lung carcinomas include adenocarcinomas, squamous cell lung carcinomas, and large cell lung carcinomas. These tumors have only a 20–30% positive clinical response, however the cause of treatment resistance is still unknown.

microRNAs (miRNAs) are evolutionarily conserved, endogenous noncoding RNA of about 22 nucleotides (nt) in length involved in protein-expression regulation at the posttranscriptional level [1]. With the advent of miRNA expression profiling, significant effort has been made to correlate miRNA expression with tumor prognosis [2], [3]. To date, a number of down-regulated miRNAs found in lung cancer correlate with patient survival [4], [5], [6] and with therapeutic response [7]. This finding led many research groups to identify the molecular mechanisms responsible for the deregulation of these miRNAs in human cancers.

Epigenetics refers to changes in gene expression that occur without alteration in DNA sequence. There are two primary and interconnected epigenetic mechanisms: DNA methylation of CpG islands within promoter regions and post-translational modification of histone tails as acetylation, phosphorylation, methylation and ubiquitilation [8], [9], [10]. In addition to known genetic mutations involved in neoplastic transformation, many evidences suggest that cancer cells have an altered epigenetic machinery since either DNA methylation or histone modifications are modified compared to normal cells [11].

Recently we found both *in vivo* and *in vitro* that miR-212 was strongly downregulated in lung cancer and that its ectopic expression increased TRAIL (tumor necrosis factor-related apoptosis-inducing ligand) sensitivity of lung cancer cells [12]. Since many microRNAs downregulated in cancer have been tightly related to CpG island hypermethylation and/or alteration in histone marks modifications [13], [14], [15] we wondered whether the same modifications could be involved in miR-212 silencing in lung cancer. Therefore, in this manuscript we investigated both DNA methylation patterns and histone modifications of miR-212 promoter region in Calu-1 (NSCLC) and MRC5 (normal human fibroblasts derived from fetal lung fibroblast) cells carrying different miR-212 expression profiles. Our results show that although the transcriptional start site of miR-212 is embedded in a CpG island, its transcriptional inactivation in lung cancer is not associated to DNA hypermethylation status but instead to a change in the methylation status of histone tails linked to the promoter region of this microRNA. Furthermore, by using tissue specimens of lung cancer at different TNM staging we analyzed the expression levels of miR-212 and found that its silencing is closely associated with the severity of the disease.

## Results

### Expression of miR-212 in different lung cancer stages

**Figure 1:**
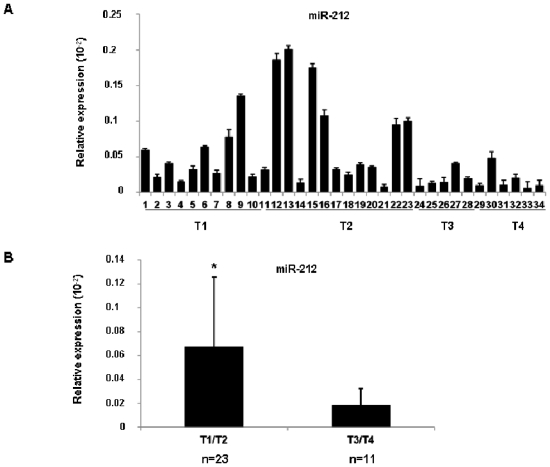
Expression of miR-212 in human lung cancer tissues specimens. (**A**) Total RNA extracted from tissue specimens collected from 34 NSCLC-affected individuals at different TNM staging (T1-T2-T3-T4) was used to analyze miR-212 expression by Real-Time PCR. (**B**) Mean ± SD of miR-212 expression of pool T1/T2 vs T3/T4. Error bars indicate SD. *p<0.05, by t test. As shown, in the T1/T2 staging the expression of miR-212 is clearly heterogeneous while in the T3/T4 staging the miR-212 expression is strongly silenced and correlates with severity of disease.

Recently we demonstrated both *in vitro* and *in vivo* that miR-212 expression in lung cancer is down-regulated compared with normal lung [12]. To test whether or not its silencing correlates with the stage of the tumor, tissue specimens were collected from 34 NSCLC-affected patients at different TNM staging (clinical features are summarized in Table 1). We then analyzed miR-212 expression by qRT-PCR (Figure 1A). As shown, in the T1/T2 samples, the expression of miR-212 is mostly heterogeneous. On the contrary, in the T3/T4 samples, miR-212 expression was homogeneously down-regulated. Figure 1B shows the average of T1/T2 samples compared to T3/T4. These data indicate that the silencing of miR-212 is closely related to the severity of the disease.

**Table 1:** Clinical features of the patients.

Patients	Lung tissue	Stage	Sex	Age
1	Squamous cellcarcinoma	T1	M	80
2	“	“	M	61
3	“	“	M	80
4	Adenocarcinoma	“	F	42
5	“	“	F	58
6	“	“	M	68
7	“	“	F	72
8	“	“	M	63
9	“	“	M	68
10	“	“	F	61
11	Squamous cellcarcinoma	T2	M	64
12	“	“	F	56
13	“	“	M	76
14	“	“	F	48
15	“	“	M	64
16	“	“	M	75
17	“	“	M	76
18	“	“	M	64
19	“	“	M	64
20	“	“	F	74
21	“	“	F	75
22	“	“	M	74
23	“	“	M	61
24	Adenocarcinoma	T3	F	55
25	“	“	M	86
26	“	“	M	74
27	“	“	M	63
28	“	“	M	64
29	Adenocarcinoma	T4	F	48
30	“	“	F	61
31	“	“	F	62
32	“	“	M	64
33	Squamous cellcarcinoma	“	M	61
34	“	“	F	56

**Figure 2:**
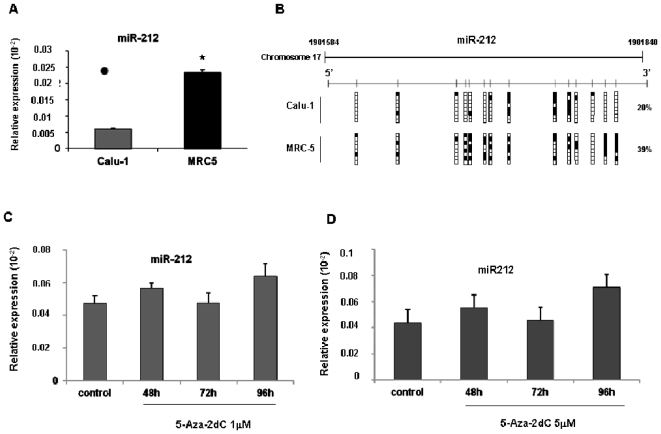
Analysis of DNA methylation status of the miR-212 promoter region. (**A**) Expression analysis of miR-212 in NSCLC (Calu-1) and normal human fibroblasts derived from fetal lung (MRC5) by Real- Time PCR. (**B**) Bisulfite genomic sequencing analyses of the miR-212 CpG island in Calu-1 and MRC5 cells. Eight single clones are represented for each sample. Black and white squares represent methylated and unmethylated CpG respectively, and each vertical bar illustrates a single CpG. (**C**) Expression analysis of mature miR-212 by Real-Time PCR in Calu-1 cells in absence (control) or in presence of 1 µM (left panel) and 5 µM (right panel) of DNA methylation inhibitor 5-aza-2′-deoxycytidine (5-Aza-2dC), as indicated. In NSCLC, DNA hypermethylation of CpG island is not detected suggesting that this epigenetic modification is not responsible of miR-212 silencing. Means ± SD of four independent experiments in triplicate are given.

Role of epigenetic on miR-212 expression- Using the CpG Island Searcher program (http://www.cpgislands.com/) we found that miR-212 locus was embedded within a CpG island. It is known that several miRNAs are epigenetically silenced in association with CpG island hypermethylation in cancer cells [16], [17]. We thus investigated whether the loss of miR-212 expression in NSCLC was associated with DNA hypermethylation. We analyzed by bisulfite genomic sequencing the DNA methylation status of the predicted transcriptional start sites (TSSs) of pri-miR-212 [18], [19] in Calu-1 and MRC5 cell lines that express different amounts of miR-212 (Figure 2A). As shown in figure 2B, in Calu-1 the region analyzed was largely under-methylated. To confirm that the transcriptional silencing of miR-212 in lung cancer was not associated with CpG methylation, Calu-1 cells were treated with two different concentrations of the DNA methylation inhibitor 5-aza-2′-deoxycytidine (5′Aza) as indicated. miR-212 expression was then evaluated by q-RT-PCR (Fig. 2C–D). As shown in Figure 2C–D, upon 5′Aza treatment, miR-212 expression levels were unchanged. Taken together these results indicate that methylation is not related to miR-212 silencing in NSCLC.

### Methylation and acetylation analysis of histones associated to the TSS of miR-212

**Figure 3:**
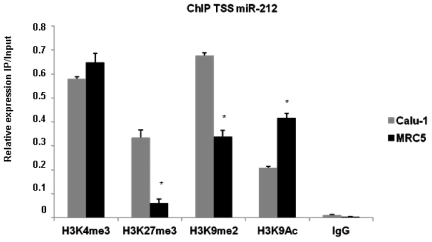
Transcriptional silencing of miR-212 in lung cancer cells is associated with histone modifications. ChiP and Real-Time PCR were used to study, in Calu-1 and MRC5 cells, histone modifications in the promoter region of miR-212 using antibodies directed against H3K4me3, H3K27me3, H3K9me2 and H3K9Ac. A negative control antibody (IgG) was included in the ChiP assay. Means ± SD of four independent experiments in triplicate are given.

We then investigated whether modifications in histone proteins could account for miR-212 downregulation in NSCLC. We performed ChiP analysis using four antibodies against specific histone modifications: H3K4me3 and H3K9Ac, generally associated with transcriptional active chromatin, and H3K27me3/H3K9me2 generally associated with transcriptional inactive chromatin. Subsequently, we compared the histone marks pattern of the predicted miR-212 TSS both in Calu-1 and MRC5 cells. As expected, we found significant differences in the methylation status of H3K27 and H3K9 and in the acetylation status of H3K9 (Figure 3). Specifically, in Calu-1 cells that express low levels of miR-212, TSS of miR-212 is enriched by the presence of histone proteins with covalent modifications involved in gene silencing (H3K27me3 and H3K9me2). On the contrary, high miR-212 expressing MRC5 cells exhibited an increase in histone modifications associated with gene activation (H3K9Ac) compared to Calu-1 cells. Our data suggest that epigenetic modifications on histones at miR-212 TSS contribute to its epigenetic silencing in NSCLC.

### Altered miR-212 expression in NSCLC is associated with changes in histone modifications

The histone methyl transferase EZH2, a specific H3K27 methyl transferase, is frequently over-expressed in human cancer [20]. Moreover, immunohistochemical analyses revealed that G9a, a specific H3K9 methyltransferase, was highly expressed in lung cancer tissue specimens compared to normal lung samples [21].

**Figure 4:**
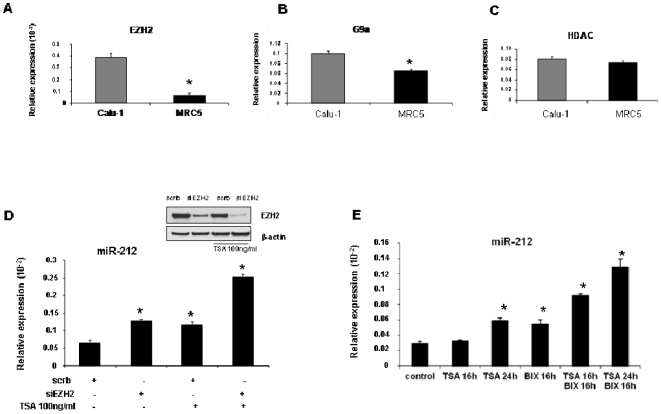
Role of EZH2 and G9a enzymes in histone methylation pattern and in miR-212 expression levels in NSCLC. (**A**) Expression analysis of EZH2, (**B**) G9a and (**C**) HDAC enzymes by Real-Time PCR in Calu-1 and MRC5 cells. (**D**) Expression analysis of mature miR-212 in Calu-1 cells untreated (−) or treated (+) with siEZH2 and TSA inhibitor or (**E**) with TSA and BIX01294 (BIX) inhibitors. (**D**) Calu-1 cells were transfected with specific EZH2-siRNA for 24 hours. Then, the cells were incubated with 100 ng/ml of TSA for 24 hours and miR-212 expression was evaluated by qRT-PCR. Down-regulation of EZH2 expression after siEZH2 transfection was evaluated by western blotting using anti-EZH2 antibody. To confirm equal loading the membrane was immunoblotted with anti-β-actin antibody. (**E**) Calu-1 cells were treated with 100 ng/ml of TSA for 16 or 24 hours in presence or in absence of 10 µM of BIX for 16 hours and miR-212 expression was evaluated by Real-Time PCR. These results suggest that in Calu-1 cells the upregulation of EZH2 and G9a enzymes contributes to increase histone methylation and thus to decrease miR-212 expression levels. Means ± SD of four independent experiments in triplicate are given.

Based on these findings, we analyzed by q-RT-PCR the expression levels of EZH2 and G9a enzymes in Calu-1 and MRC5 cells. As shown in Figure 4, Calu-1 cells exhibited increased amounts of EZH2 (A) and G9a (B) compared to MRC5 cells. On the contrary, expression levels of H3K9 de-acetylase HDAC did not differ in the two cells type Figure 4C.

The same results were obtained in another NSCLC cell line, A549. As shown in figure S2, A549 cells show low levels of miR-212 (A), upregulation of EZH2 (B) and G9a (C) and similar levels of HDAC (D), comparing with MRC5.

In order to confirm the involvement of histone modifications in miR-212 silencing in NSCLC, we inhibited both EZH2 expression and HDAC activity and then analyzed the effects on miR-212 expression in Calu-1 cells. To this aim, Calu-1 cells were transfected with EZH2-siRNA and/or treated with TSA, a specific inhibitor of HDAC. miR-212 was then evaluated by q-RT-PCR. As expected, either siRNA-mediated knock-down of EZH2 methylase, or TSA-mediated inhibition of HDAC de-acetylase activity contributed to increase miR-212 expression levels (Figure 4D). Interestingly, the effect was greater (four-folds) in the presence of both inhibitions. The same effect was observed in A549 cells (Figure S2E). The inhibition of EZH2 and HDAC produced an increase (two-folds) of miR-212 also in MRC5 cells (Figure S2F).

In order to determine the role of G9a on miR-212 silencing, Calu-1 cells were treated with BIX01294, a specific inhibitor of G9a methylase, in the presence or in absence of TSA. miR-212 was evaluated by q-RT-PCR. As expected, the treatment with both reagents, contributed to increase miR-212 expression levels up to 5 folds (Figure 4E). Taken together these results suggest that the upregulation of EZH2 and G9a enzymes in lung cancer contributes to increase histone methylation and thus to decrease miR-212 expression levels. When used at the same time, siEZH2/TSA or BIX01294/TSA produced greater effects on miR-212 expression compared to the single incubation. Thus, it is possible to speculate that H3K27me3 or H3K9me2-mediated silencing machinery requires HDAC activity, as reported previously by Lachner M *et al* [22].

### Effects of inhibition of EZH2, G9a and HDAC on histone proteins

**Figure 5:**
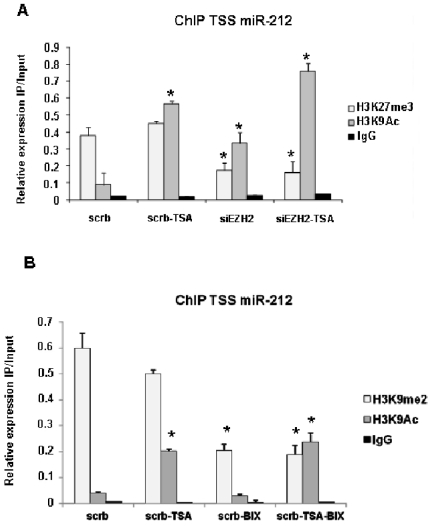
Inhibition of EZH2, G9a and HDAC enzymes cause a change in the histone marks pattern at the TSS of miR-212. ChiP and qRT-PCR were used to analyze, in Calu-1 cells, histone modifications at the predicted miR-212 TSS (**A**) in absence (scrb) or in presence of TSA and siEZH2, and (**B**) in absence (scrb) or in presence of TSA and BIX inhibitors, respectively. (**A–B**) Calu-1 cells were treated in presence of the indicated inhibitors as described in the legend of figure 4. The results indicate that the histone modification catalyzed by EZH2, G9a and HDAC enzymes sites are involved in miR-212 silencing in NSCLC. Means ± SD of four independent experiments in triplicate are given.

Based on this findings, we wondered whether the inhibition of EZH2, G9a and HDAC enzymes in Calu-1 cells may cause a change in the H3K27me3, H3K9me2 and H3K9Ac histone marks pattern at the TSS of miR-212 affecting thus miR-212 expression levels. To this aim, Calu-1 cells were treated with siEZH2 and TSA and ChiP analyses were performed using H3K27me3 and H3K9Ac antibodies, respectively. Interestingly, inhibiting both methylase (EZH2) and de-acetylase (HDAC), strongly increased the acetylation of H3K9 and significantly decreased the methylation of H3K27 of miR-212 TSS compared with untreated cells (Figure 5A). These results correlate with the up-regulation of miR-212 in the same experimental condition (compare Figure 4D with Figure 5A). In addition, Calu-1 cells were treated with BIX01294 and TSA inhibitors and ChiP analyses were carried using H3K9me2 and H3K9Ac antibodies, respectively. As expected, we found that inhibiting the catalytic activity of either methylase (G9a) or de-acetylase (HDAC), strongly decreased the methylation of H3K9 and significantly increased the acetylation of H3K9 on miR-212 TSS compared with untreated cells (Figure 5B). Taken together, these results strongly demonstrate that specific changes in histone modifications determine miR-212 silencing in NSCLC.

### Effect of inhibition of EZH2, G9a and HDAC on apoptosis in Calu-1 cells

**Figure 6:**
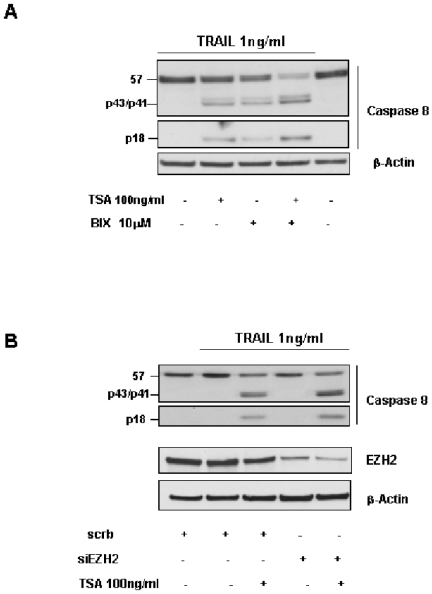
Inhibition of G9a and HDAC enzymes induced TRAIL sensitivity. (**A**) Calu-1 cells were treated in absence (−) or in presence (+) of 100 ng/ml of TSA and/or 10 µM of BIX, as previously described, then the cells were incubated with 1 ng/ml of Super-Killer TRAIL for 3 hours. Lysates were analyzed by western blotting with anti-caspase-8 antibody. Cleavage of caspase-8 was more evident in Calu-1 cells treated in presence of both inhibitors. (**B**) Calu-1 cells were transfected with siEZH2 and treated in absence or in presence of 100 ng/ml TSA, as previously described, then cells were incubated with 1 ng/ml of TRAIL. β-actin antibody was used as loading control.

It is known that inhibition of HDAC by TSA, induces cell cycle arrest and apoptosis in different cancer cells as glioma, bladder cancer, leukemic cells and SCLC [23], [24]. Besides, in human bronchoepithelial cells, transfection of G9a and EZH2 by siRNAs induced apoptosis and G1 arrest, respectively [25]. Our recent findings demonstrated that ectopic expression of miR-212 sensitizes Calu-1 cells to TRAIL-induced apoptosis [12]. We therefore verified whether the inhibition of EZH2, G9a and HDAC enzymes may increase TRAIL sensitivity of Calu-1 cells. To this aim, Calu-1 cells were treated with BIX01294 and TSA to inhibit G9a and HDAC enzymes and apoptosis was evaluated by western blot analysis of caspase-8 activation upon TRAIL treatment. As reported in Figure 6A, caspase-8 is clearly activated in presence of G9a or HDAC inhibition. Moreover, caspase-8 activation further increases in the presence of both enzymes inhibition, condition that correlates with up-regulation of miR-212 (compare Figure 6A with Figure 4E). For the same purpose, apoptosis induction was evaluated by inhibiting the EZH2 and HDAC enzymes by siEZH2 and TSA. Differently from the results obtained above, caspase-8 was activated only in presence of HDAC inhibition and not in presence of EZH2 knock-down (Figure 6B). To further confirm these results we performed Annexin V apoptosis assay (Figure S1).

## Discussion

MicroRNAs are small non-coding RNAs molecules that function as endogenous posttranscriptional silencers of target genes and are important regulators of different cellular processes including apoptosis [26], [27]. Defects in the apoptotic program may contribute to treatment resistance and tumor progression, and may be caused by deregulated expression of anti-apoptotic molecules. The number of discovered microRNAs and their targets grow quickly indicating their essential role in maintaining gene expression profile. We recently demonstrated for first time the pro-apoptotic role of miR-212 in lung cancer. Specifically, we found that miR-212 was able to target the anti-apoptotic protein PED upregulated in lung cancer [28] suggesting that its action may contribute to tumor suppression since it negatively inhibits a molecule involved in apoptosis resistance [12]. Moreover, analyzing human tissues specimens of normal and lung cancer we found that the up-regulation of PED protein in lung cancer tissues was correlated with miR-212 silencing and *in vitro* with apoptosis resistance. Nevertheless, the relationship between miR-212 silencing with progression and severity of disease was still unknown. To this end, using tissue specimens from NSCLC-affected patients in the T1-T2-T3 and T4 staging, we found that in T1/T2 the expression of miR-212 was heterogeneous whereas in the T3/T4 all samples analyzed showed miR-212 downregulation. These results suggest that silencing of miR-212 in lung cancer is closely related to the severity of the disease. However, the molecular mechanisms involved in miR-212 silencing in lung cancer are still unknown, and this was the focus of our study.

Several studies have analyzed the expression of miR-212 in different cells types in order to determine its role in tissue-specific physiology. miR-212 expression levels were found higher in forebrain regions of the adult rat brain [29] strongly downregulated in the tissues of fetuses with anencephaly [30] and, in association with miR-132, higher in hippocampal neurons. Moreover, miR-212 has been described as involved in normal dendrite maturation in newborn neurons in the adult hippocampus [31].

To date, few reports show an involvement of miR-212 in cancer but all of them indicate that this miRNA is deregulated in different human cancer. In particular, miR-212 expression decreases in both gastric carcinoma (GC) cells and in human primary GC tissues suggesting that its downregulation may be related to gastric carcinogenesis through its target genes such as methyl-CpG-binding protein [32]. In pancreatic adenocarcinoma tissues miR-212 is over-expressed and targets the retinoblastoma tumor suppressor [33] Using microarray analysis thirteen miRNA, including miR-212, were found significantly overexpressed in oral tumors [34]. In addition, increased expression of HB-EGF (heparin-binding EGF-like growth factor) due to down-regulation of miR-212 in head and neck squamous cell carcinoma (HNSCC) has been described as a possible mechanism of cetuximab resistance [35].

Cancer cells have a specific epigenome. In the last decade many studies have shown that the aberration of epigenetic marks such as DNA methylation in CpG islands and covalent modifications of histone proteins involved in chromatin organization, contribute significantly to malignant transformation. On the other hand, it has been demonstrated that the expression of miRNAs can be affected by epigenetic changes. In bladder tumors it was found that miR-127 is silenced by promoter methylation and its expression could be restored by hypomethylating agents [16]. Lujambio *et al* treated several cancer cell lines derived from lymphonode metastases with DNA demethylating agents and using miRNA expression microarray analysis found that miR-148a, miR-34b/c, and miR-9 family showed cancer specific CpG island hypermethylation [36]. Similarly, in colon cancer cells miR-124a silencing was associated with CpG island hypermethylation [17]. Using bioinformatics programs we found a CpG island in the promoter of miR-212 gene. Aberrant methylation of the promoter CpG island region is a common epigenetic mechanism for transcriptional silencing. Therefore, we measured the effects of AZA on the transcriptional regulation of miR-212 in Calu-1 cells that express low levels of miRNA. Surprisingly, although the promoter element of miR-212 resides within a CpG island we found that Calu-1 treatment with specific inhibitor for DNA methylase did not change the expression of miR-212.

Epigenetic silencing in mammalian cells is mediated by at least two distinct histone modifications: H3K27 trimethylation and H3K9 dimethylation [37]. The relationship between DNA hypermethylation and these histone modifications is not completely understood. Interestingly, Kondo *et al* showed a newly identified epigenetic aspect of gene deregulation in cancer cell [38]. They found that up to 5% of the genes on the CpG microarray were silenced in cancer cells by marked elevation in H3K27me3 associated with relatively low levels of promoter DNA methylation. In addition, it was recently found that miR-22 silencing involves accumulation of H3K27me3 independent of promoter DNA methylation in acute lymphoblastic leukemia [39]. These data suggest that H3K27me3 is an alternate mode of gene silencing in cancer independent from DNA methylation. Consistent with these recent studies we performed ChIP analysis in lung cancer cells (Calu-1) and in normal human fibroblasts derived from fetal lung (MRC5) to investigate changes of histone modifications associated with transcriptional active and/or inactive chromatin of miR-212 promoter region. As expected, we found increasing amounts of H3K27me3 and H3K9me2 (covalent modification involved in gene silencing) in the promoter region of miR-212 in Calu-1 compared to MRC5 cells and increasing amounts of H3K9Ac (covalent modification involved in gene expression) in the promoter region of miR-212 in MRC5 compared to Calu-1 cells. Moreover, we found that inhibitors of the enzymes involved in histone modifications, i.e. siEZH2, TSA and BIX01294, were able to up-regulate miR-212 expression in Calu-1 cells. Taken together these results indicate that, although the DNA hypermethylation appears to be essential for gene silencing, the transcriptional silencing of miR-212 in NSCLC involves H3K27me3/H3K9Ac or H3K9me3/H3K9Ac-associated histone modification independent of promoter DNA methylation.

We also tested the effects on TRAIL-induced cells death in NSCL of the different inhibitors.

In fact, our recent findings demonstrated that ectopic expression of miR-212 sensitizes Calu-1 cells to TRAIL-induced apoptosis [12]. Our data show that TRAIL sensitivity of Calu-1, assessed by the western blot analysis of caspase 8 and by FACS analysis, was greater increased by the inhibition of G9a then that of EZH2. The absence of the effect of siEZH2 on TRAIL sensitivity in Calu-1 cells may be ascribed to different mechanisms. It is possible that: (i) the 70% reduction of EZH2 obtained by the treatment with the specific siRNA is not sufficient to increase TRAIL response (ii) EZH2 knock-down, besides miR-212, may regulate other important signaling molecules involved in TRAIL signaling (iii) to regulate TRAIL- signaling pathway, EZH2 may need the concomitant HDAC inhibition that may modulate the expression levels of different molecules involved in TRAIL response. Further studies are needed to explore these different hypothesis.

In conclusion, this study shows that miR-212 silencing is correlated to the severity of the disease since it is significantly down-regulated in T3/T4 staging rather than in T1/T2 staging and that its silencing in lung cancer is associated to histone modifications rather than DNA hypermethylation.

## Materials and Methods

### Cell culture

Calu-1 (NSCLC) and MRC5 (normal human fibroblasts derived from fetal lung fibroblast) cells were grown in DMEM. Media were supplemented with 10% heat-inactivated fetal bovine serum (FBS), 2 mM L-glutamine and 100 U/ml penicillin/streptomycin.

### Lung cancer samples

A total of 34 Formalin-Fixed, Paraffin-Embedded (FFPE) tissue samples consisting of both adeno and squamous cell carcinomas were collected from the archives of the Department of Pathology, University Hospital of Kuopio, Finland. Permission to use the material was obtained from the National Supervisory Authority for Welfare and Health of Finland and the study was accepted by the ethical committee of the Northern Savo Hospital District, Kuopio, Finland.

### RNA extraction and Real-Time PCR

#### Cell culture

Total RNAs (miRNA and mRNA) of Calu-1 and MRC5 cells was extracted using miRNeasy mini Kit (Qiagen) according to the manufacturer's protocol. *Tissue specimen:* Total RNA (miRNA and mRNA) from FFPE tissue specimens was extracted using RecoverAll Total Nucleic Acid isolation Kit (Ambion) according to the manufacturer's protocol. Reverse transcription of total miRNA and mRNA was performed using 500 ng (from cell culture) or 50 ng (from FFPE tissue specimens) of total RNA/sample by miScript Reverse Transcription Kit (Qiagen) according to the manufacturer's protocol. Quantitative analysis of miR-212, EZH2, G9a and HDAC were performed by RealTime PCR using miScript SYBR Green PCR Kit (Qiagen) according to the manufacturer's protocol. As internal reference we used RNU5A and GAPDH. EZH2 except (Fw: CCACCATTAATGTGCTGGAA Rv: TTCCTTGGAGGAGTATCCACA) all primers used were purchased from Qiagen. The reaction for detection of miRNA and mRNAs was performed as previously described [12]. Experiments were carried out in triplicate for each data point, and data analysis was performed by using software (Bio-Rad).

### DNA Methylation analysis

The CpG Island Searcher program (http://www.cpgislands.com/) was used to establish that miR-212 coding region is embedded in a CpG island. DNA methylation status was evaluated by sequencing analysis of bisulfite-modified genomic DNA included in the corresponding CpG island. Bisulfite treatment induces chemical conversion of unmethylated cytosine into uracil leaving methylated cytosine unchanged. Genomic DNA was modified using EZ DNA Methylation Kit (Zymo Research) according to the manufacturer's protocol. Bisulfite-treated DNA was used as template to amplify the region including miR-212 transcription start site on reverse strand using the following primers: 5′-TTTTGGGTGGTATTTGAATTTT-3′; RV 5′-CCCCTCCTCAATTCCTAAA-3′. Then, PCR products were cloned into p-GEM-T Easy Vector System II (Promega), and eight independent clones from each sample were sequenced.

### Chromatin Immunoprecipitation analysis

Chromatine Immunoprecipitation assays were carried out using Lowcell ChIP Kit (Diagenode) according to the manufacturer's protocol. Briefly, the cells were treated with 1% formaldehyde, a cross-linking agent, for 10 min. Then, chromatin was sheared with a Bioruptor (Diagenode) to an average length of 0.4–0.8 kb. Sheared chromatin was immunoprecipitated for 16 hours at 4°C using the following antibodies: anti-thrimethyl-K4 histone H3, anti-thrimethyl-K27 histone H3, anti-dimethyl-K9 histone H3 and anti-acetyl-K9 histone H3 (Diagenode). In addition, 1/100 of the solution collected before adding the antibody was used as an internal control for the amount of input DNA. Real-Time PCR was carried out in 25 µl containing 5 µl of the immunoprecipitated DNA, 12.5 µl SYBR green PCR Master mix (Qiagen) and 150 nM of specific primers (FW 5′-GGAGTCCAGCTTCCTCTCCT-3′; RV 5′-GCTCCTGGGGGTCTTCAC). The PCR protocol entailed 10 min at 95°C and 40 cycles of 15 sec at 94°C and 1 min at 60°C. Experiments were carried out in triplicate for each data point, and data analysis was performed by using software (Bio-Rad).

### Small Interfering RNA Transfection

The siRNA duplex for negative control and EZH2 mRNA (Dharmacon) were transiently transfected for 48 h in Calu-1 cells using LIPOFECTAMINE 2000 (Invitrogen), according to the manufacturer's instructions. Then, expression values of EZH2 and miR-212 levels were evaluated by Real-Time PCR and western blot analysis. Experiments were carried out in triplicate for each data point.

### Protein isolation and Western blotting

Cells were washed twice in ice-cold PBS, and lysed in JS buffer (50 mM HEPES pH 7.5 containing 150 mM NaCl, 1% Glycerol, 1% Triton ×100, 1.5 mM MgCl2, 5 mM EGTA, 1 mM Na3VO4, and 1× protease inhibitor cocktail). Protein concentration was determined by the Bradford assay (Biorad) using bovine serum albumin as standard, and equal amounts of proteins were analyzed by SDS-PAGE (12.5% acrylamide). Gels were electroblotted onto polyvinylidene difluoride membranes (Millipore, Bedford, MA). For immunoblot experiments, membranes were blocked for 1 hr with 5% non-fat dry milk in Tris Buffered Saline (TBS) containing 0.1% Tween-20, and incubated at 4°C over night with primary antibody. Detection was performed by peroxidase-conjugated secondary antibodies using the enhanced chemiluminescence system (Amersham-Pharmacia Biosciences). Primary antibodies used were: anti-EZH2 (Cell Signaling), anti-caspase 8 (Cell Signaling), and anti-βactin (Sigma).

### Cell death assessment by Annexin V staining

Cell death was assessed by annexin V and FACS analysis as previously described [12]. Briefly, cells were seeded at 12×10^4^ cells per 60-mm dish, grown overnight in 10% FBS/DMEM, washed with PBS, then transfected with siEZH2 or a scrambled sequence. After 24 hours cells were treated with of 100 ng/ml of TSA for 24 hours and/or 10 µM of BIX for 16 hours. Following incubation, cells were washed with cold PBS and removed from the plates by very mild trypsinization conditions (0.01% trypsin/EDTA). The resuspended cells were washed with cold PBS and stained with FITC-conjugated annexin V antibody and propidium iodide (PI) according to the instructions provided by the manufacturer (BD Pharmigen, San Diego, CA). Cells were then subjected to flow cytometric analysis. The percentage of apoptosis indicated was corrected for background levels found in the corresponding untreated controls.

### Statistical analysis

Continuous variables are expressed as mean values ± standard deviation (SD). One and two-tailed student's t-test were used to compare values of test and control samples. p<0.05 was considered significant.

## Supporting Information

Figure S1 **Effects of epigenetic modifications on cell death.** Calu-1 cells were treated in absence (−) or in presence (+) of 100 ng/ml of TSA and/or 10 µM of BIX, or transfected with siEZH2 and treated in absence or in presence of 100 ng/ml TSA, as previously described. The cells were incubated with 1 ng/ml of Super-Killer TRAIL for 3 hours. Apoptosis was evaluated with Annexin V staining. A representative dot blot is shown (**A**). Means ± SD of four independent experiments in triplicate are given (**B**).
(EPS)

Figure S2 **Effects of EZH2 and HDAC inhibition on miR-212 expression in A549 and MRC5 cell lines.** Expression analysis of miR-212 (**A**), EZH2 (**B**), G9a (**C**) and HDAC (**D**) by Real-Time PCR in A549 and MRC5 cells. Expression analysis of mature miR-212 in A549 (**E**) and MRC5 (**F**) cells untreated (−) or treated (+) with siEZH2 and TSA. Cells were transfected with specific EZH2-siRNA for 24 hours, then incubated with 100 ng/ml of TSA for 24 hours and miR-212 expression was evaluated by qRT-PCR. Down-regulation of EZH2 expression after siEZH2 transfection was evaluated by western blotting using anti-EZH2 antibody. To confirm equal loading the membranes were immunoblotted with anti β-actin antibody. Means ± SD of four independent experiments in triplicate are given.
(EPS)
